# The Design and Implementation of Kerberos-Blockchain Vehicular Ad-Hoc Networks Authentication Across Diverse Network Scenarios †

**DOI:** 10.3390/s24237428

**Published:** 2024-11-21

**Authors:** Maya Rahayu, Md. Biplob Hossain, Samsul Huda, Yuta Kodera, Md. Arshad Ali, Yasuyuki Nogami

**Affiliations:** 1 Graduate School of Environmental, Life, Natural Science and Technology, Okayama University, Okayama 700-8530, Japan or biplob.eee@kyau.edu.bd (M.B.H.); yuta_kodera@okayama-u.ac.jp (Y.K.); yasuyuki.nogami@okayama-u.ac.jp (Y.N.); 2 Electrical Engineering Departement, Politeknik Negeri Bandung, Bandung 40559, Indonesia; 3 Electrical and Electronic Engineering Department, Khwaja Yunus Ali University, Sirajganj 6751, Bangladesh; 4 Green Innovation Center, Okayama University, Okayama 700-8530, Japan; shuda@okayama-u.ac.jp; 5 Faculty of CSE, Hajee Mohammad Danesh Science and Technology University, Dinajpur 5200, Bangladesh; arshad@hstu.ac.bd

**Keywords:** Vehicular Ad-Hoc Network, Kerberos authentication, blockchain

## Abstract

Vehicular Ad-Hoc Networks (VANETs) play an essential role in the intelligent transportation era, furnishing users with essential roadway data to facilitate optimal route selection and mitigate the risk of accidents. However, the network exposure makes VANETs susceptible to cyber threats, making authentication crucial for ensuring security and integrity. Therefore, joining entity verification is essential to ensure the integrity and security of communication in VANETs. However, to authenticate the entities, authentication time should be minimized to guarantee fast and secure authentication procedures. We propose an authentication system for VANETs using blockchain and Kerberos for storing authentication messages in a blockchain ledger accessible to Trusted Authentication Servers (TASs) and Roadside Units (RSUs). We evaluate the system in three diverse network scenarios: suburban, urban with 1 TAS, and urban with 2 TASs. The findings reveal that this proposal is applicable in diverse network scenarios to fulfill the network requirements, including authentication, handover, and end-to-end delay, considering an additional TAS for an increasing number of vehicles. The system is also practicable in storing the authentication message in blockchain considering the gas values and memory size for all scenarios.

## 1. Introduction

The Intelligent Transportation System (ITS) is recognized as a key element in the future transportation model, playing a crucial role in shaping an advanced transportation network in the age of Digital Transformation (DX) [1]. An ITS itself has a key element that enables communication between vehicles and the road infrastructures, known as VANET. The main goal of such communication is to convey vital messages encompassing factors such as speed, location, trajectory, and urgent notifications indicating dangerous situations [2]. These potential capabilities impact efficiency and management improvement of traffic flow. This improvement is influenced by providing information to vehicles, optimizing their routes, and reducing traffic congestion. The potential of the capabilities resides in augmenting efficiency and overseeing traffic flow through the dissemination of current information to vehicles, optimizing their pathways, and reducing traffic congestion [3]. Additionally, VANETs facilitate efficient emergency response, prompt incident documentation, and coordinated evacuation plans during critical situations. Utilizing VANET scalability and adaptability provides numerous benefits that can enhance “the security and effectiveness of transportation networks”.

**Figure 1:**
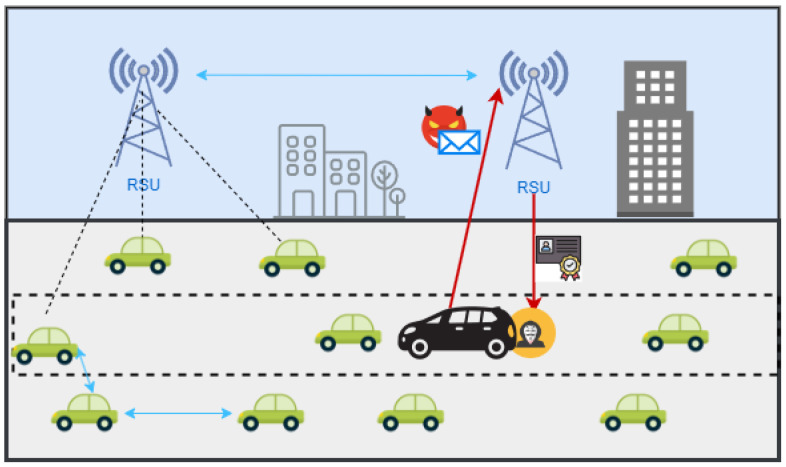
The vulnerability of VANET.

Nevertheless, due to the open nature of VANETs, high node mobility, instability, and constantly changing network topology, security becomes a major concern when implementing VANETs. Within VANETs, vehicles demonstrate high velocities and recurrent disconnections, rendering them particularly vulnerable to potential attacks from adversaries seeking to acquire or manipulate valuable data within the network [4]. As shown in Figure 1, the attacker can send deceptive messages to the network or ask for the credential data of other users from the network. The blue lines indicate communication between vehicles or between RSUs, the dotted lines show vehicle-to-infrastructure communication and the red lines indicate dangerous information sent or received from a malicious node.

To guarantee the security of Vehicular Ad-Hoc Networks (VANETs) and its various applications, particularly those related to safety, it is essential to verify the legitimacy of transmitted messages and the identities of their sources. Failure to do so could allow unauthorized vehicles to spread false information or engage in nefarious activities undetected, posing serious risks to transportation infrastructures and the safety of individuals using the roads. Consequently, the implementation of an authentication protocol in VANETs is imperative to thwart malevolent message transmission by adversaries and safeguard crucial network information [5].

The efficiency of message authentication in VANETs is crucial for maintaining safety. Any delay in authentication could lead to outdated or unreliable information being used to make driving decisions, potentially causing accidents. Quick authentication ensures that messages are verified rapidly, allowing for immediate action based on accurate and timely information. Delays in authentication can lead to a lag in the dissemination of critical safety information, increasing the risk of accidents due to delayed driver reaction times. Ensuring that authentication processes are efficient and meet the low latency requirements of VANET safety applications is essential to maintain the effectiveness and reliability of these systems in enhancing road safety. Most of the safety applications in VANETs exhibit low latency, typically around 100 ms. Consequently, there is a need for a proficient mechanism to validate incoming messages within a brief timeframe of 100 ms, prior to transmitting a fresh safety notification [6].

Traditional methodologies for ensuring secure and authenticated message propagation, primarily relying on message encryption and key administration, are restricted to ensure secure message interchange solely among identified source and destination pairs. Due to the dynamic topology of VANETs, these methods cannot be directly implemented in these vehicular networks. The dissemination of messages within VANETs is also susceptible to insider assaults (i.e., assaults originating from authenticated VANET participants) that have the potential to breach the confidentiality of disseminated messages or disseminate malevolent messages. Consequently, the preservation of message integrity and authenticity during transmission within VANETs has emerged as a critical concern [7].

The Kerberos protocol functions as a robust authentication mechanism specifically crafted to enhance security within networks that are susceptible to breaches, utilizing session keys for transmitting data during the signaling phase as opposed to the principal key [8]. However, the centralized structure of the Kerberos authentication mechanism brings forth added complicacy, which could lead to extended authentication delay and add complications to the transition process due to the participation of numerous entities.

In the field of VANETs, many current solutions rely on a central trusted authority, which is not a scalable solution and becomes the network’s single point of failure. To address these issues, researchers introduce a decentralized blockchain-based authentication solution for VANETs that integrates blockchain with VANETs. This ensures the distributed structure and preserves an immutable ledger of data, strengthening the system’s integrity for VANETs. The Inter Planetary File System (IPFS), Ciphertextbased Attribute Encryption (CP-ABE), and the Ethereum blockchain are the foundations for the distributed VANET system suggested in [9].

Several pieces of research have been conducted to create secure VANET authentication, as shown in Table 1. A Two-Factor Lightweight Privacy-preserving (2FLIP) scheme has been introduced in an authentication method for Vehicular Ad-Hoc Networks (VANETs), utilizing a decentralized certificate authority and a biological-password-based, two-factor authentication to significantly reduce computation and communication overhead while ensuring strong privacy preservation and resilience against denial-of-service attacks [10]. In [11], the authors design a protocol to ensure Secure and Efficient Message Authentication (SEMA) between vehicles and Roadside Units (RSUs), focusing on preventing vehicles from being falsely accused and ensuring robustness against various security attacks. It reaches that goal through a combination of pseudonym-based and group-based methods. SEMA achieves mutual authentication between vehicles and RSUs, ensuring that the communication is not only secure but also respects the privacy of the participants. In [12], it introduces a secure and efficient authentication protocol specifically designed for Vehicle-to-Vehicle (V2V) communication within Vehicular Ad-Hoc Networks (VANETs) and Internet of Vehicle (IoV) technologies, aiming to enhance traffic system management and road safety. A novel feature of the protocol is the vehicle password change phase, which incorporates the use of a honey list technique to thwart offline password-guessing attacks, enhancing the overall security of the system. However, those three articles [10,11,12] still utilize the centralized network, which can create a single point of failure, and the exchanged data can be altered or tampered with, leading to potential trust issues.

To overcome those issues, several researchers have applied blockchain technology during the VANET authentication phase. In [13], the authors build a new blockchain-based authentication infrastructure for wireless networks that utilizes AES, the Temporal Key Integrity Protocol (TKIP), and the Counter Mode with Cipher Block Chaining Message Authentication Code Protocol (CCMP) to secure the user’s login information, using blockchain to verify user credentials. It utilized the hyperledger fabric blockchain in its proposed method. In [14,15,16], the authors combine the ECC and blockchain certificate management to make a secure and decentralized authentication in VANETs. However, among all the proposed authentication methods that utilize blockchain, every paper utilizes and collects the data from either the network or the blockchain environment only, and none of them utilize data from both the network and the blockchain side.

In our proposal, we integrate Kerberos authentication and blockchain to conduct the innovational authentication system for VANETs. This approach stores Kerberos authentication messages in the blockchain’s distributed ledger that can be accessed in the Trusted Authentication Server (TAS) and all RSUs. This authentication message storing aims to simplify handover delay processes, shorten authentication delay, and securely keep the authentication message. To further improve system performance, we implement Kerberos using AES-128 encryption instead of the original Data Encryption Standard 77 (DES77) [17] aiming to reduce authentication time. Then, we assess the feasibility of blockchain technology for VANET authentication scenarios using Ethereum and simulate the process with Omnet++.

To evaluate its effectiveness, we have designed three distinct scenarios. The first scenario involves simulating the system in a suburban environment within the Tsushima Campus area at Okayama University, Japan. This simulation involves 100 vehicles and one Trusted Authority Server (TAS). The second scenario involves an urban environment in the Okayama Station area, featuring a higher vehicle density with 200 vehicles and one TAS. Then, the third scenario is a variation of the second but includes an additional TAS, totaling two TASs. In our evaluation, we focus on network performance metrics such as authentication delay, handover delay, and end-to-end delay. Additionally, we assess blockchain performance by measuring factors such as gas usage and the memory size of the blocks.

The contributions of this paper are listed below:

- It proposes a system that enhances the effectiveness of the authentication protocol by storing the authentication message from Kerberos authentication in the blockchain system.
- It reduces the authentication delay by utilizing blockchain to access the authentication message, which can make the re-authentication process simpler than the initial authentication process.
- It evaluates the authentication delay, handover delay, and end-to-end delay to compute the performance of the system and investigate the effects of TAS on these delays.
- It evaluates the gas value and the memory size of the block to store the authentication message, which ensures the practicability of the system if implemented in the blockchain environment.
- It evaluates the proposed method in diverse networks, including the suburban area and the urban area.

## 2. Preliminaries

This section provides a theoretical exposition of VANETs, coupled with an annotations table, elucidation of Kerberos authentication, and blockchain framework employed in this study. Moreover, this chapter explores the intricacies of establishing secure communication in VANETs.

### 2.1. Vehicular Ad-Hoc Network

Vehicular Ad hoc Networks (VANETs) represent a system of mobile vehicles through which operators within automotive settings are able to access instantaneous data, sophisticated traffic management, and event distribution. The primary objectives of VANETs include ensuring secure driving conditions, optimizing traffic movement, and reducing the occurrence of accidents. Generally, the architecture of VANETs comprises three primary elements: vehicles, Road-Side Unit (RSU), and Trusted Authority Server (TAS) [4]. Every vehicle is embedded with an On-Board Unit (OBU), which has the role of effectively supporting the process of transmitting and receiving the wide spectrum of messages that are in circulation within the network. This pivotal function enables the establishment of a continuous and uninterrupted flow of communication as well as the seamless exchange of data among the various vehicles that are interconnected within the intricate framework of this dynamic vehicular network. VANETs exhibit several characteristics, such as high node mobility, dynamic network topology, real-time transmission constraints, and constrained computation and storage capabilities. V2V utilizes the 5.9-GHz DSRC protocol, enabling cars in VANETs to exchange traffic-related data with neighboring vehicles every 100 to 300 ms. Regarding RSU, the communication range for each RSU ranges from 1 to 3 km based on the IEEE 802.11p wireless communication protocol [9]. Interactions among these entities involve transmitting and receiving crucial messages, encompassing periodic, event-triggered, and emergency messages.

#### Annotation Table

This subsection delineates various annotations and abbreviations for all terms presented in this manuscript, encompassing entities, keys, message names, and contents. The comprehensive list of abbreviations and annotations can be found in Table 2.

### 2.2. Kerberos Authentication

Kerberos employs symmetric key cryptography to provide a secure authentication mechanism for both the vehicle and Roadside Units (RSUs). The utilization of a session key for crucial procedures, including those involving the Ticket-Granting Server (TGS) and specific services, distinguishes Kerberos authentication as a notable protocol. The decryption of messages that carry session keys and other significant information is achieved through the utilization of the entities’ confidential keys.

The original authentication mechanism utilized in Kerberos V5 [18] can be outlined as the following process:

(1) $$\begin{array}{cc} RA= & ID_{v}||Realm_{v}||ID_{TGS}||Times||Nonce_{1}\left|\right|PreAuth. \end{array}$$

(2) $$\begin{array}{cc} RP= & ID_{v}||TGS||E(K_{vs},[K_{TGS}||Times||Nonce_{1}||Realm_{TGS}||ID_{TGS}]). \end{array}$$

### 2.3. Blockchain Technology

A blockchain is composed of a sequence of interconnected blocks that contain transaction details and are linked through cryptographic methods to uphold the integrity of data. The transaction information is permanently stored in immutable blocks that are distributed among all network participants. The utilization of decentralization aims to address inconsistencies in the majority of data, supported by a consensus mechanism that ensures the consistency of the ledger [19]. This mechanism plays a crucial role in upholding the security and reliability of the entire data network. Transaction details are spread across multiple blocks, which are then grouped together to establish a verified chain of data. Each block’s contents are hashed and securely preserved in the subsequent block. The development of an additional cryptography algorithm is necessary for integration into the blockchain network [20]. Subsequently, this off-chain environment interacts with the on-chain blockchain environment to leverage the cryptographic algorithm features. Nowadays, Hyperledger Fabric and Ethereum have been developed as well-known examples of blockchain platforms. These platforms represent tangible expressions of the rising inclination towards the enhanced accessibility and acceptance of these advanced and revolutionary technologies in a variety of sectors spanning industries and fields of study. Ethereum is specifically selected as the fundamental platform for blockchain-based development in this research, owing to its compatibility with decentralized Applications (dApps) and situations that emphasize transparency and openness.

## 3. The Proposed Method and Scenarios

This chapter elucidates the overview of the authentication system, organized as a sequence of system phases in VANETs. The components encompass the suggested system framework, commencement, enrollment, and authentication steps.

### 3.1. Overview of the System

This section delineates the outlines of the proposed system, comprising its entity functions, an overview of the steps, and an overview of the authentication and blockchain part. The methodology integrates Kerberos authentication with the blockchain framework for the preservation of vehicles’ authentication messages [21]. By employing blockchain technology, a possible decrease in handover time can be achieved as a result of its decentralized characteristics. Following the vehicle’s completion of the initial authentication phase, there is no need to establish a connection with the Kerberos server when transitioning to the handover phase. The functions of entities, an overview of the phases, and an explanation of the authentication part and blockchain part are presented in the following sub-sections.

#### 3.1.1. Entities and Functions

Our system has three main entities: Trusted Authority Server (TAS), Road Side Unit (RSU), and vehicle. TAS is a Kerberos server that registers all entities, authenticates vehicles, and uploads authentication messages to the blockchain. The TAS has sub-parts, including an Authentication Server (AS) and a Ticket Granting Server (TGS). RSUs are the bridge between vehicles and the TAS. They enable vehicles to enter the network, support data transfer among the network entities, and broadcast information related to traffic conditions. The vehicles play a role as network nodes and data sources. They are equipped with an On-Board Unit (OBU) for transmitting road-related information to RSUs and other surrounding vehicles.

#### 3.1.2. Overview of the Phases

**Figure 2:**
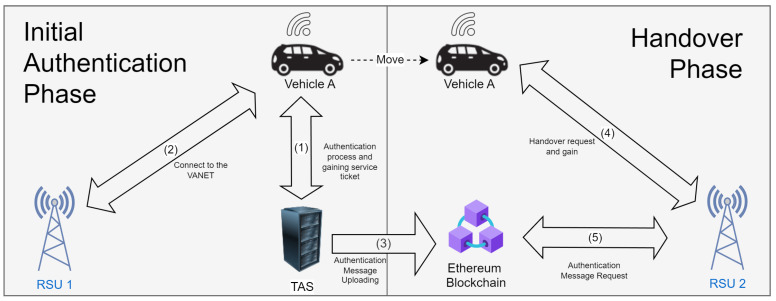
Resume of initial authentication phase and handover process.

The phases in the overall system include the system initialization and registration phase, initial authentication phase, and handover phase. The registration phase has a function to register all of the entities, including the RSU, TAS, and vehicles. The initial authentication phase and handover phase are presented in Figure 2. In the initial authentication process, vehicles send the authentication request to the TAS, especially to the AS. If verified, the AS sends the ticket-granting ticket to the vehicles as a credential to obtain the Service Ticket from the TGS. With that service ticket, the vehicle is authenticated and can connect to the network through RSU1. While authenticating the vehicle, the TAS will generate the authentication message as the credential to the vehicle to perform the re-authentication in the handover phase. When leaving the RSU1 communication range, the vehicle will send the handover request to RSU2 by sending its authentication message. RSU2 has to check the credentials to match the data from the blockchain. RSU2 sends the authentication message request to the blockchain, and if it matches the vehicle will be re-authenticated. In this handover process, the vehicle does not need to perform the initial authentication phase anymore, which can reduce the authentication delay.

#### 3.1.3. Main Parts of the Kerberos-Blockchain VANET System

**Figure 3:**
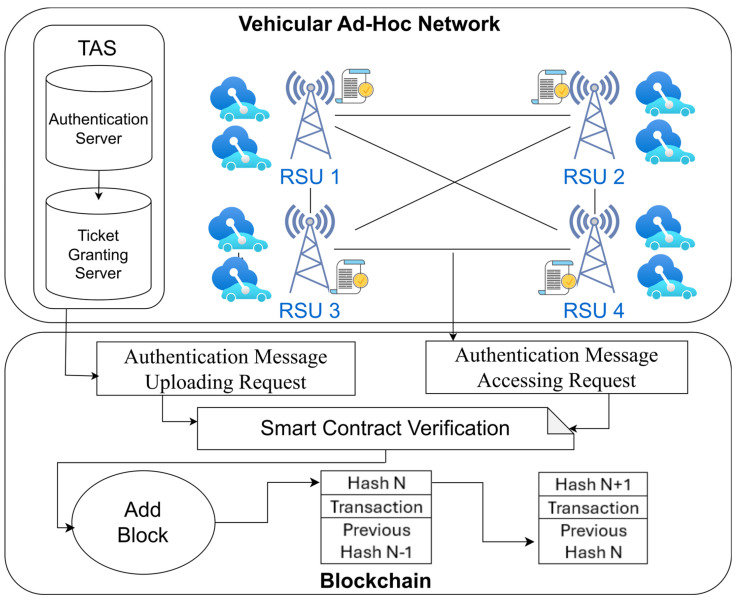
Main parts of the Kerberos-blockchain VANETs system.

This research includes two main parts: the authentication part and the blockchain part, as shown in Figure 3. The authentication part uses Kerberos authentication. The Kerberos server has two sub-servers that have different functions. The Authentication Server (AS) has the function of checking the vehicle’s credentials and generating a TGT as the vehicle’s credential to be able to obtain service tickets from the Ticket Granting Server (TGS). The TGS will also generate an authentication message as the credential in the handover process that needs re-authentication for every vehicle that enters the new RSU communication range. That authentication message will be uploaded to the blockchain.

The blockchain part includes smart contract creation to upload and access that authentication messages. The agreements that we used to write down the solidity codes are entity name, entity ID, and network name. The entities include vehicles, RSU, and TAS. Within these solidity codes, we created four smart contract functions, including the deployment function, the entity registration, the authentication message uploading and, the authentication message uploading function. The deployment function is a function at the beginning to initialize the system. Entity registration is a function to register every entity in the system. Authentication message uploading is a function for uploading the authentication messages, and authentication message accessing is a function to access the authentication messages. In the authentication message uploading and accessing function, we need to know the entity name, which we create as a unique parameter. Based on this parameter, we set the entities that are able to upload the authentication messages as only the TAS. Then, the entity that can access the authentication messages is the RSU. After entering the input parameter, it will be verified by all the other parties in the network. After reaching a consensus, the transaction will be added as a new block in the blockchain.

To implement the system, we use Ethereum blockchain. Blockchain is particularly suitable for VANETs (Vehicular Ad-Hoc networks) for several reasons, primarily due to its robust and well-proven characteristics. There are several existing Distributed Ledger Technologies (DLTs) besides blockchain, such as Directed Acyclic Graph (DAG), Holochain, and Hashgraph [22]. While each of these DLTs offers innovative features, they come with disadvantages that make them less suitable in VANETs compared to blockchain. DAG is relatively newer and has shown vulnerabilities, such as potential attacks exploiting low transaction fees and double-spending. These are significant risks in a critical environment like VANETs. Holochain, Radix, and Corda are newer technologies and do not have the same level of standardization, while many blockchain platforms, especially Ethereum, have established standards and protocols that can be leveraged for VANET applications. While Corda supports smart contracts, it is more business-oriented, focusing on enterprise use cases rather than the real-time, high mobility of VANETs. The support for smart contracts in blockchain, especially in Ethereum, allows the creation of automated services such as vehicle coordination and cooperative agreements, fitting well with the VANET’s requirements. The ethereum use the standard version f3553dd [23]. There are several platforms of blockchain that have already been established and have matured standardization, such as Ethereum and Hyperledger [24]. This system utilizes the Ethereum blockchain rather than Hyperledger. Although it supports smart contracts, Hyperledger emphasizes chain code, which has limited flexibility compared to Ethereum’s smart contracts, which have robust and flexible capabilities. It enables more complex vehicle interactions and autonomous services, essential in VANET systems.

### 3.2. Testing Scenarios

**Figure 4:**
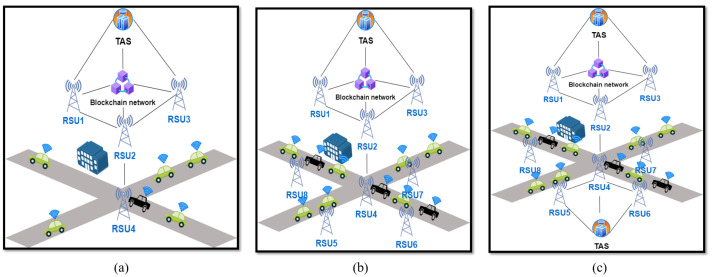
Experiment case scenarios: (**a**) suburban, (**b**) urban with 1 TAS, and (**c**) urban with 2 TASs.

**Figure 5:**
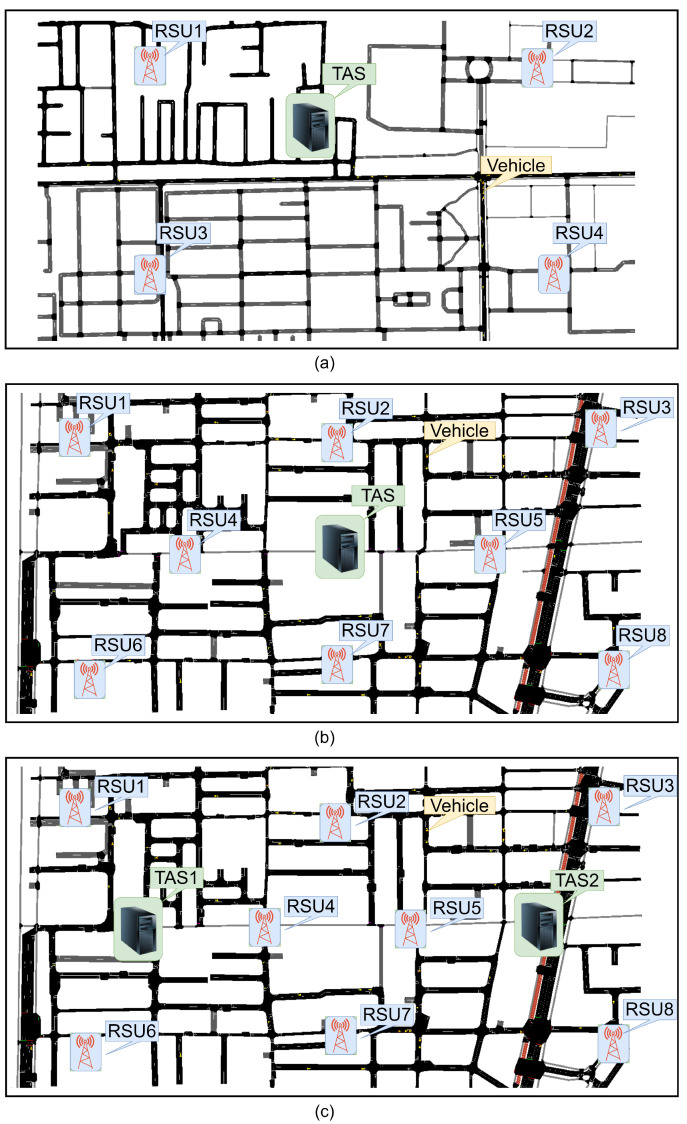
Maps for the scenario of (**a**) suburban and (**b**) urban with 1 TAS and (**c**) urban with 2 TASs.

Figure 4 shows three case scenarios for the experimentation of the system. The first scenario, as shown in Figure 4a, focuses on a suburban area. In this scenario, we utilized 100 vehicles, four Roadside Units (RSUs), and one Traffic Analysis System (TAS). The limited number of RSUs is justified by the relatively low density of the suburban environment, which does not necessitate a large number of vehicles. The experiments were conducted using the Tsushima Campus area maps in Okayama, Japan, as shown in Figure 5a. The maps are generated by using Openstreet Maps, licensed under the Open Database License (ODbL) and inserted into SUMO tools to simulate the VANET. SUMO is distributed under the Eclipse Public License (EPL), which is a permissive open-source license. In the first scenario, the TAS is placed in the middle of the network, surrounded by RSU1, RSU2, RSU3, and RSU4.

The second scenario is an urban scenario with 1 TAS, as shown in Figure 4b, characterized by higher vehicle density. In this case, we increased the number of vehicles from 100 to 200. Given the greater availability of infrastructure in the city, we also doubled the number of Roadside Units (RSUs) from 4 to 8. This scenario was conducted in the densely populated area around Okayama Station in Okayama City, Japan, as highlighted in Figure 5b. The objective is to determine whether or not the performance of the authentication system remains within acceptable limits when the number of vehicles increases. Specifically, we aimed to assess if the authentication delay in the VANET environment stays below the critical threshold of 100 milliseconds, as stipulated in [6]. We placed the TAS in the centre of the maps, surrounded by the 8 RSUs. The small yellow dots in the maps indicate the vehicles.

The third scenario, depicted in Figure 4c, was also implemented using the urban area maps depicted in Figure 5c. In this case, we employed more than one Trusted Authority Server (TAS) to ensure the system’s performance in an environment requiring additional infrastructure. Specifically, we utilized 8 RSUs, 200 vehicles, and 2 TAS units. The introduction of multiple TAS units aimed to distribute the authentication management tasks effectively, accommodating the increased number of vehicles. In the third scenario, the TASs are positioned on the right and left sides of the map, unlike the central placement in scenarios 1 and 2, to achieve balanced load distribution. This configuration is intended to prevent load imbalances that could negatively impact TAS performance.

The number of vehicles in a VANET system varies based on factors like area size, use case, and simulation goals. Denser urban areas have higher vehicle counts, while less populated areas have lower values. In one study [25], the authors chose 50–200 vehicles to evaluate routing protocols in a city scenario with high-density traffic. In a separate analysis [26], authors selected 0–120 vehicles to study urban traffic congestion and system effectiveness. In another study [27], authors chose 10–100 vehicles to simulate low and moderate traffic scenarios in real-world vehicular environments. This range helps analyze how increasing vehicle density impacts key performance metrics such as packet delivery ratio and delays. The selection of vehicle numbers for simulations depends on balancing real-world traffic patterns with available computational resources.

### 3.3. System Initialization and Registration Phase

Offline enrollment comprises the provision of credentials from the vehicles, encompassing their unique vehicle identification number, user identification number, password, origin, destination, type of service, and permissions (read, write, and modify). Roadside Units (RSUs) are also enrolled with their specific location, MAC address, IP address, and RSU ID. Prior to commencing the verification process, the Kerberos server will produce and transmit the confidential keys for each entity.

### 3.4. Initial Authentication Phase

**Figure 6:**
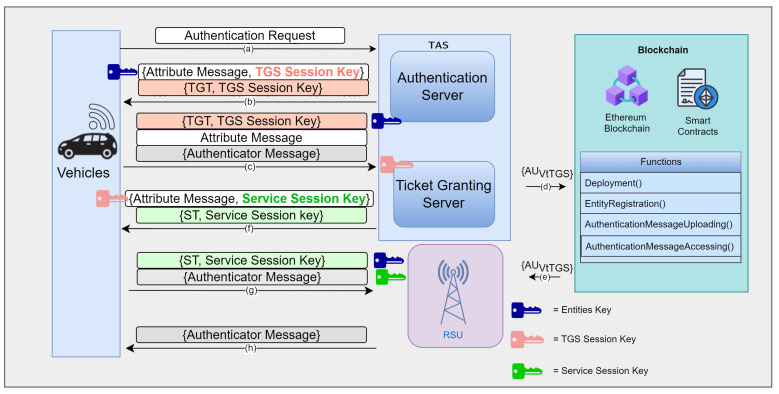
Initial authentication phase.

The protocol for the authentication phase comprises numerous distinct stages. These stages are delineated as the communication between the vehicle and the Authentication Server (AS), the communication between the vehicle and the TGS, the phase dedicated to storing authentication messages on the blockchain, and the communication stages between the vehicle and the RSU. The illustration of the signaling process during this authentication phase can be observed in Figure 6.

#### 3.4.1. Vehicle and AS Communication Stage

This phase encompasses communications transmitted from the vehicle to the AS, and vice versa, mirroring steps (a) and (b) depicted in Figure 6. Initially, the vehicle transmits the Registration Acknowledgment (RA) to the AS, containing the $ID_{v}$, the specific service name that the vehicle intends to utilize (in this instance, the service name pertains to RSU service), $IP_{v}$, and the stipulated duration for the Ticket Granting Ticket (TGT) ($Req_{TGT}$). The $Req_{TGT}$ serves to restrict the duration, thereby enhancing system security through a finite time constraint. These data will be forwarded to the AS in the Trusted Authentication Server (TAS).

The messages that are sent by the vehicles to the AS are shown in Equation (3). Equations (4) and (5) show the corresponding response messages. The parenthesis symbol denotes one group of unencrypted messages, while the bracket symbol illustrates the encrypted messages.

(3) $$\begin{array}{c} RA=(ID_{v}||ID_{s}||IP_{v}||Req_{LT}). \end{array}$$

(4) $$\begin{array}{ccc} AT_{AStV}= & K_{vs}[ID_{TGS}||TS_{AStV}\left|\right| & LT_{TGT}\left|\right|K_{TGSse}]. \end{array}$$

(5) $$\begin{array}{ccc} TGT= & K_{TGSs}[(ID_{v}||ID_{TGS}||TS_{TGT}\left|\right| & IP_{v}||LT_{TGT}\left|\right|K_{TGSse})]. \end{array}$$

The Authentication Server (AS) possesses a registry of authorized users alongside their corresponding confidential keys. Verification involves confirming the presence of $ID_{v}$ and the messages in the aforementioned registry. Upon successful validation, a duplicate of $K_{Vs}$ is extracted. Subsequently, the AS initiates the creation of an $AT_{AStV}$, following which the Ticket Granting Ticket (TGT) is dispatched to the user. $AT_{AStV}$ incorporates $ID_{TGS}$, timestamp $TS_{AStV}$, and a designated duration of validity. The TGT includes $ID_{v}$, $ID_{TGS}$, $TS_{TGT}$, $IP_{v}$, and $LT_{TGT}$. Encryption of both messages is carried out using $K_{TGSse}$, a symmetric key generated randomly, intended for the user’s decryption of various communications from the Transportation Authority Server (TAS) and Road Side Unit (RSU) as a service server, limited to that specific instance. The Attribute message ($AT_{VtTGS}$) is encrypted with $K_{Vs}$, while the TGT undergoes encryption with $K_{TGSs}$. Subsequently, these two messages are dispatched from the AS to the vehicle.

#### 3.4.2. Vehicle and TGS Communication Stage

The decryption of $AT_{AStV}$ by the key $K_{Vs}$ is necessary for the vehicle, followed by the acquisition of $K_{TGSse}$. Subsequently, two messages will be generated by the vehicle, with the initial message comprising $ID_{S}$ and $LT_{TGT}$. The second message, the $AU_{VtTGS}$, includes $ID_{v}$ and $TS_{VA}$. Encryption of the $AU_{VtTGS}$ is carried out using $K_{TGSse}$. This key uses the AES encryption that utilizes the 128-bit key length. The 128-bit AES is lightweight and suitable to be implemented in the VANET environment rather than AES 192-bit and AES 256-bit. The VANET has several resource constraints, including low power consumption and memory efficiency. Vehicles in a VANET environment may rely on embedded systems with limited power. AES-128 strike a balance between security and power consumption. The AES-128 also requires less memory and processing than AES with longer key lengths, which is advantageous in devices with limited memory, such as those in vehicular communication systems. Moreover, our system also utilizes blockchain, which has a limited block size. This particular phase is illustrated in step (c) within Figure 6. After generating the $AU_{VtTGS}$, the TGS will sent it to the blockchain network to store it in step (d). Then, the $AU_{VtTGS}$ is sent to the RSU in step (e). The details of $AU_{VtTGS}$ storage in the blockchain is explained in Section 3.5.

In addition to the aforementioned generated messages, a vehicle is set to transmit the TGT acquired from the AS. Subsequently, the vehicle will dispatch the trio of messages illustrated in Equations (6)–(8) to the TGS. Upon receipt, the TGS will verify the presence of $ID_{S}$ in plaintext within its own registry. The $ID_{S}$ should be found in the TGS server’s records; the TGS will duplicate the $K_{Ss}$. Encoded within the TGT lies a $K_{TGSse}$, which enables the TGS to decipher the $AU_{VtTGS}$. Those messages will be sent to the vehicles in step (f).

(6) $$\begin{array}{ccc} TGT= & K_{TGSs}[(ID_{v}||ID_{TGS}||TS_{TGT}\left|\right| & IP_{v}||LT_{TGT}\left|\right|K_{TGSse})]. \end{array}$$

(7) $$\begin{array}{c} AT_{VtTGS}=(ID_{s}||Req_{LT}). \end{array}$$

(8) $$\begin{array}{c} AU_{VtTGS}=K_{TGSse}[ID_{v}||TS_{VA}]. \end{array}$$

#### 3.4.3. Vehicle and RSU Communication Stage

Upon reception of attribute messages and the service ticket, the system will progress to steps (g) and (h) of Figure 6. The vehicles will utilize $KT_{GSse}$ to decipher the $AT_{TGStV}$, subsequently obtaining $K_{Sse}$. Subsequently, a fresh $AU_{VtS}$ will be created by the vehicle, encompassing $ID_{v}$ and $TS_{VtS}$, which will then be encrypted using $K_{Sse}$. The vehicle will then transmit both the ST and the authentication message to the server. In this scenario, the RSU represents the desired service for the vehicles. The messages sent from the vehicle to the RSU are detailed below:

(9) $$\begin{array}{ccc} ST= & K_{Ss}[(ST||ID_{v}||ID_{S}||TS_{TGStV}\left|\right| & IP_{v}||LT_{ST}\left|\right|K_{Sse})]. \end{array}$$

(10) $$\begin{array}{c} AU_{VtS}=K_{Sse}[ID_{v}||TS_{VtS}]. \end{array}$$

(11) $$\begin{array}{c} AU_{Sm}=K_{Sse}[ID_{v}||TS_{StV}]. \end{array}$$

The RSU will perform a similar procedure involving the TGS. Subsequently, the RSU will decipher the ST utilizing its $K_{Ss}$, thereby acquiring authorization from the $K_{Sse}$. This authorization will be utilized to decrypt $AU_{VtS}$. Upon completion, the RSU will generate its own $AU_{Sm}$ comprising $ID_{RSU}$ and the $TS_{StV}$, as demonstrated by Equation (11).

Subsequently, the $AU_{Sm}$ will be delivered to the user and subsequently decrypted by the vehicle utilizing $K_{Sse}$. The vehicle will then proceed to verify whether or not the service name included in the authentication corresponds to its designated service recipient. To mitigate potential replay assaults, the vehicle will also validate the timeliness of the authentication by inspecting $TS_{StV}$. Furthermore, the vehicle will maintain its cache mechanism. After the mutual authentication procedure between the user and the service, the vehicle will retain a protected version of the service ticket in its cache for future reference.

#### 3.4.4. TAS and RSU Interaction

TAS and RSU has a non-direct interaction in the initial authentication phase and the handover process. In the initial authentication phase, as shown in Figure 6, it indicates that the TAS and RSU do not directly communicate; however, their functions are interrelated. The TAS comprises two components: the Authentication Server (AS) and the Ticket Granting Server (TGS). The AS is primarily responsible for managing the initial authentication of vehicles requesting RSU services within the secure network. Its functions include verifying vehicle identities, issuing Ticket Granting Tickets (TGTs), and encrypting user credentials for security. The TGS then issues service tickets, which vehicles use to access RSU services. The service server validates these tickets to authenticate the client and authorize service access. In the handover process, the TGS generates the $AU_{VtTGS}$, serving as the credential required during the procedure. This credential is uploaded to the blockchain, which is managed via a smart contract to control the uploading and access of the $AU_{VtTGS}$. The smart contract’s Solidity code includes parameters such as entity name, entity ID, and network name. The TAS is the designated entity authorized to upload the $AU_{VtTGS}$. This credential is accessed by the RSU when a vehicle submits a handover request. The RSU retrieves the $AU_{VtTGS}$ by entering its entity name, which is verified through a consensus mechanism. Upon validation, the transaction is added as a new block, granting the RSU access to the $AU_{VtTGS}$.

### 3.5. Authentication Message Uploading in the Blockchain Phase

In the TGS communication stage, after obtaining the authentication message ($AU_{VtTGS}$), the TGS will send it to the blockchain. When authenticating a vehicle, this $AU_{VtTGS}$ proves the authenticity of the car. The solidity code is used to obtain this $AU_{VtTGS}$, which was produced previously by the off-chain environment (VANET). After that, to see it on the Ganache platform, it must pass via the smart contract. In this case, AuthenticationMessageUploading() is used to take the $AU_{VtTGS}$ as input and then pass it as an argument of the function. After that, the entity “TAS” sends an AuthenticationMessageUploading() transaction request to store the $AU_{VtTGS}$ in the blockchain. It will be saved in the blockchain block once the transaction is successful. After that, by establishing the events inside the function, we can see the information extracted from the blockchain on the Ganache platform.

Here, we consider the entity “TAS” as a sender and “RSU” as a receiver. We also use “EntityID” equal to “1” and “2” for the “TAS” and “RSU”, respectively. Both entities exist in the same network as “TsushimaVanet”. To obtain the $AU_{VtTGS}$ from the blockchain, the entity “RSU” now delivers an AuthenticationMessageAccessing() transaction request. It will be able to acquire the $AU_{VtTGS}$ from the blockchain after completing the transaction.

### 3.6. Handover Phase

**Figure 7:**
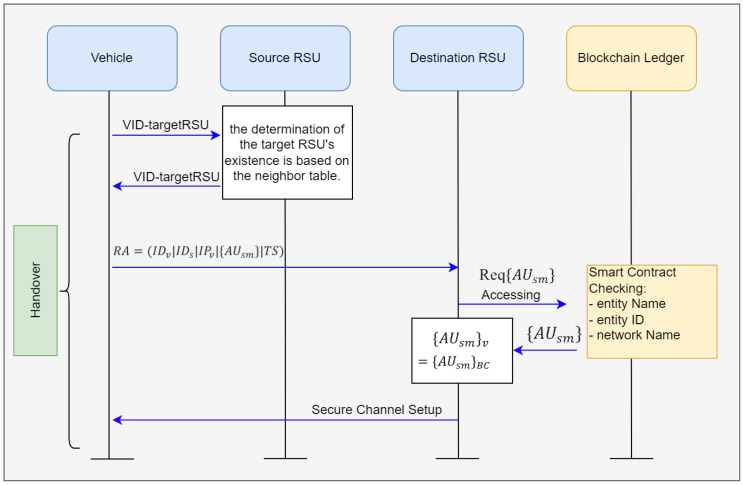
Handover signaling procedure.

The schematic representation illustrating the handover signaling process of the suggested approach can be observed in Figure 7. Upon recognition by a preceding Road Side Unit (RSU) that a vehicle or a cluster of vehicles has exited its designated coverage zone, an initiation of the handover procedure takes place.

- Step 1: The vehicle send the VID-targetRSU that verifies the target RSU has authenticity. The source RSU will determine the authenticity of the destination RSU by checking the neighbor table.
- Step 2: The vehicle advances by transmitting a request message to the destination RSU. This RSU will send the request for the AuthenticationMessageUploading() transaction to the blockchain ledger.
- Step 3: The blockchain ledger will check the RSU with the smart contract agreements, especially for the entity name. After that, it will give the $AU_{s}m$ to the destination RSU.
- Step 4: That RSU will equalize the authentication message sent by the vehicle and the authentication message in the blockchain ledger. Following successful validation, the destination RSU sends a message confirming the completion of the handover to the vehicle, thereby finalizing the transfer. Subsequently, the vehicle updates its ledger and disseminates the information.

## 4. Implementation and Discussion

The following section describes the simulation conditions and the outcomes of the suggested system, including network performance and blockchain performance results.

### 4.1. Implementation Environments

The evaluation of the proposed system’s feasibility is detailed in this section. Table 3 presents the deployment context, framework, requisite technical resources, and software components.

**Figure 8:**
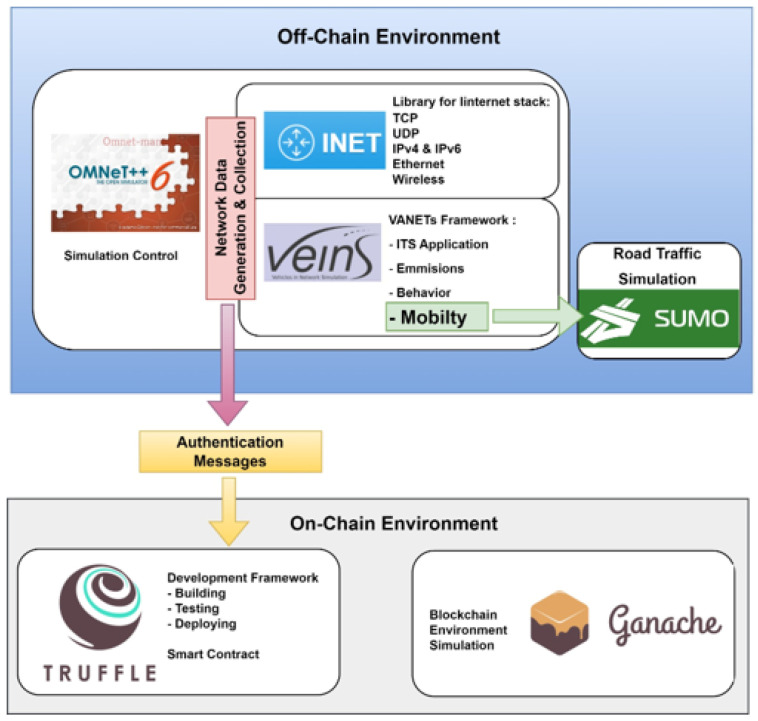
Off-chain and on-chain environment of the proposed system.

To develop the proposed system, various tools are employed to model the processes among the entities in the vehicular network. We utilize the off-chain and on-chain environment that is illustrated in Figure 8. In this case of an off-chain environment, we use Omnet++ and SUMO to simulate the VANET authentication protocol, and to generate the authentication messages. Omnet++ is utilized in our experiment as a tool for modeling and simulating discrete systems [28]. The design of the vehicular network uses NED language, combined with VEINS and INET frameworks. VEINS is an open-source framework designed for vehicular network simulations, utilizing OMNeT++ and SUMO [29]. The INET Framework serves as an open-source model library for OMNeT++, providing various protocols and models for communication networks [30].

The setup of vehicle nodes, as well as the creation of maps, junctions, and routes, are achieved through the use of SUMO [31]. After generating the authentication messages are sent to the on-chain environment. In the on-chain environment, we run the blockchain and set the solidity code for the smart contract. In our experiment, we use Truffle and Ganache in the on-chain environment. Truffle constitutes a comprehensive development environment designed specifically for the Ethereum blockchain, offering an array of tools that facilitate the construction, evaluation, and deployment of smart contracts [32]. Ganache is the Ethereum development tool to simulate a blockchain environment locally and to conduct tests on the deployed smart contracts [33].

The relevant parameters that are considered for the experiment are detailed in Table 4. The communication model that we used is the IEEE 802.11p standard [34] that is specifically designed for vehicular environments, supports vehicle-to-vehicle and vehicle-to-infrastructure communication, and is compatible with Dedicated Short-Range Communication (DSRC).The simulation area we used is 5000 m × 2500 m, which is located in two different areas with a scale of 1:20,000. In scenario 1, which represents a suburban area, we considered the geographical area near Okayama University, Tsushima, Japan. The initial creation of entities in the first stage comprises 100 vehicles, 4 RSUs, and 1 TAS, which functions like a Kerberos server. In scenarios 2 and 3, which represent an urban area, we used an area near Okayama Station that has higher vehicle density. Scenario 2 includes 1 TAS, 200 vehicles, and 8 RSUs. Scenario 3 contains 2 TASs, with the same number of vehicles and RSUs. We chose a communication range of 1000 m for the RSUs to allow broader coverage with fewer RSUs, and this range is often used as a standard in VANETs that aligns with typical ranges used in the IEEE 801.11p standard. The communication range of the vehicle that we used is 100 m, which provides reliable communication without excessive interference. The data rate set in the simulation is 27 Mbps, which aligns with the capabilities of the IEEE 802.11p standard. The safety messages are sent every 30 s to reach a sufficient update rate for traffic and hazard information. The mobility that we used in the simulation is the Duaroute Mobility as a standard mobility that is used in the IEEE 802.11p standard. Duaroute calculates routes for vehicles based on demand and network conditions. It generates routes by considering factors like road networks, traffic lights, and traffic density.

### 4.2. The Network Performance Result

This section highlights the simulation results based on the network performances. The delays for the transmission and processing of messages and also the signaling overhead are described here in detail. The scenarios of suburban and urban are considered to evaluate the performances of the network.

#### 4.2.1. Delay

**Figure 9:**
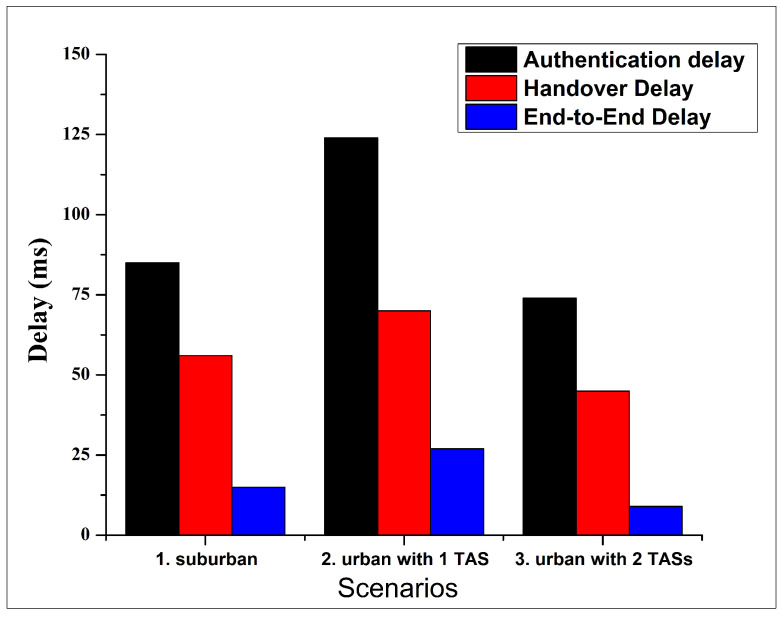
Comparison of several delays of different scenarios.

To evaluate the behavior of the VANET protocol in terms of delay, Figure 9 has been plotted. AODV is used as the routing protocol with respect to suburban, urban with 1 TAS, and urban with 2 TASs scenarios. In VANETs, there are several parameters that are directly related to delays of the system. These includes simulation area, the vehicle’s communication range, number of intermediary nodes (hops), number of vehicles and TASs, etc.

If the density of the vehicles is more in a simulation area, it leads to more data and authentication requests within the network. As a result, the demand for authentication services rises, potentially increasing the overall network delay.

The communication range of a vehicle denotes the farthest distance for direct communication with another vehicle. In a larger communication range, vehicles can communicate over greater distances without requiring intermediate nodes. As a result, this can reduce the number of hops needed to transmit data across the network, which reduces the overall network delay, as more hops can increase the overall delay due to the additional processing and transmission times at each hop.

In a VANET, the relationship between the number of vehicles, TASs, and network delay are crucial. The TAS manages the authentication and security of the system. More TASs can help to distribute the load and handle requests more efficiently, potentially reducing network delays. In the same vehicle’s communication range, if the number of TASs is fixed, then the delay of the system only depends on vehicle quantity. An increase in vehicle count correlates with a rise in system delay. We analyze three types of delay that happened in those three scenarios: authentication delay, handover delay, and end-to-end delay.

The authentication delay encompasses both the transmission and processing time of messages, starting from when a vehicle sends the Request Authentication (RA) message until it receives the $AU_{Sm}$ as the credential to enter the network through the RSU [35]. The authentication delay must not exceed 100 ms to satisfy VANET criteria [6]. Equation (12) quantifies the delay, with $T_{SRA}^{i}$ indicating the duration for vehicle authentication requests. Vehicle *i* is assigned time $T_{RFA}^{i}$ for receiving the message of service authentication from the RSU. The variable *N* signifies the total vehicle count in the simulation context [36]. The following is a breakdown of how the authentication delay is calculated:

(12) $$\mathrm{AuthDelay}=\frac{1}{N}\sum_{i=1}^{N}\left(T_{SRA}^{i}\;-\;T_{RFA}^{i}\right).$$

Figure 9 shows the delays, including the authentication, handover, and end-to-end delay for overall scenarios. In the suburban scenario, it shows that in a simulation involving 100 vehicles within a 5000 m × 2500 m communication area, the authentication delay becomes 85 ms (<100 ms), indicating that this delay performance still meets the requirement for VANETs [6]. However, when we tried to increase the number of vehicles from 100 to 200, considering the urban area, it effects the authentication delay of the network. In the urban area, we first considered 1 TAS. In this case, the authentication delay exceeds the VANET requirement and it reached 124 ms. This is because an increase in vehicles typically raises the demand of authentication services, potentially increasing the overall network delay, including authentication delay. Adding more TASs can mitigate this issue. For this, we considered 2 TASs in the same urban area without changing the other parameters and observed the effect of TASs in the overall network delay. In this case, the authentication delay reduces to 74 ms, which also meets the requirement for VANETs. This is because an additional TAS can help to distribute the load more effectively. Even when the authentication requests rise due to the increase of vehicles, this additional TAS can handle requests more efficiently throughout the network, reducing the delay associated with data transmission and processing.

Another delay observed in our study is the handover delay. Handover delay is quantified from the vehicle’s exit from the previous RSU’s coverage to its re-authentication by the new RSU [37]. The data indicate that, in every scenario, the handover delay is consistently lower than the initial authentication delay. However, the handover delay values exhibit a linear relationship with the changes in the authentication delay, increasing or decreasing correspondingly. In this proposed method, the handover delay indicates the re-authentication process associated with transitioning between RSU coverage areas; the vehicle needs re-authentication to make sure it has the correct credentials. From all of our experiments, the handover delay for scenarios 1, 2, and 3 are 55 ms, 69 ms, and 44 ms, respectively. Due to the authentication delay requirements, all the scenarios in our experiments still have values below the maximum limit (100 ms) of authentication delay. This indicates that our system functions well in the case of handover delay in all the cases, including the suburban and the urban areas.

The period required for a packet to traverse from its origin to its target location is termed end-to-end delay [38]. This is established by evaluating the time the packet leaves the source vehicle and the time it reaches the destination vehicle. Equation (13) delineates the method for accurately calculating the end-to-end delay, denoted as EED, with TA representing arrival time and TS representing sending time [39]. Based on the ETSI TS 122 186 criteria regarding the service specifications for upgraded V2X scenarios [40], the maximum permissible end-to-end delay for information interchange between an On-Board Unit (OBU) and a Roadside Unit (RSU) while platooning must be less than 20 ms. Figure 9 illustrates that scenarios 1 and 3 exhibit end-to-end delays of 15 ms and 9 ms, respectively, which meet this requirement. Conversely, scenario 2 records a 27 ms delay, surpassing the acceptable limit. This suggests that the urban scenario with 200 vehicles necessitates more than 1 TAS to meet the ETSI standard’s ETSI TS 122 186 [40] end-to-end delay requirement.

(13) $$\mathrm{EED}=\sum T_{A}\;-\;T_{S}.$$

In this study, the vehicle serves as the source node while the TAS acts as the destination node. The delay includes propagation, transmission, queuing, and processing delays. Additionally, the end-to-end delay is consistently less than the authentication delay and handover delay. The variation patterns in the end-to-end delay observed in our proposed system closely follow the authentication delay values due to their intrinsic correlation. The authentication delay is derived from the Kerberos authentication process, which includes the processing time at each node and the time taken to exchange multiple messages among entities. So, the total delay in authentication within our system is notably affected by the end-to-end delay.

In an urban setting with 2 TASs scenario (scenario 3), it shows that all types of delay are reduced compared to the other two scenarios. This reduction is attributed to the increased number of TASs. However, the reduction of the delay is not exactly 50% due to the addition of 2 TASs compared to the scenario with 1 TAS. It is only 40.3%. This discrepancy is influenced by the Ad-Hoc On-Demand Distance Vector (AODV) routing protocol utilized in the VANET system. It is a popular routing protocol for VANETs that only establishes routes when needed [41]. This protocol has the ability to promptly adjust to dynamic alterations within the network, facilitated by its on-demand route discovery mechanism, enabling swift responses to network modifications. The AODV protocol selects the minimal path based on node count between source and destination. In our authentication scheme, the sender is the vehicle and the receiver is the TAS. When the network has two TASs, one TAS may become more heavily loaded than the other, leading to load imbalance. The reason for this is that the vehicle’s hop distance to the TAS depends on its mobility and dynamic network topology. Although AODV can dynamically adjust routes if the current route becomes invalid during its path maintenance stage, the newly adjusted route may still not account for equal load balancing. This load imbalance can result in higher authentication delays. Several factors contribute to the increased authentication delays, such as load imbalance, including server processing time and queuing delay. Each authentication request requires time for the server to process, which involves the encryption and decryption of Kerberos authentication messages, message generation, and other authentication steps. If the number of authentication requests exceeds the processing time capacity, the time needed to handle the request will increase. On the queuing delay side, if one server accepts many requests at the same time, then the request has to be in the queue until the server needs to process it. The larger the queue, the more time is required for each request, thereby increasing the overall authentication delay in the TAS with higher authentication requests.

A summary of the simulation network prerequisites is illustrated in Table 5. The “✔” symbol indicates that the delay has fulfilled the network performance requirement, and the “×” symbol indicates that the delay has not fulfilled the network performance requirement. The data presented in the table indicate that scenarios 1 and 3 fulfill all the network requirements for authentication delay, handover delay, and end-to-end delay. This means that our proposal is still appropriate for implementation with 1 TAS for 100 vehicles and 2 TASs for 200 vehicles. However, scenario 2 did not fulfill the network requirements for authenticating end-to-end delay when the number of vehicles is 200 and the number of TASs is only 1. Our proposal is appropriate for implementation in the suburban area with 1 TAS and the urban area, which has more density with 2 TASs.

#### 4.2.2. Signaling Overhead

The signaling overhead parameter quantifies the signaling messages as the expense per temporal unit during which the vehicle engages in the handover process. This parameter is computed by the product of the distance to the Road Side Units (RSUs), the unit transmission overhead, and the size of the messages utilized in vehicular communication. We evaluate the signaling overhead by counting our system’s signaling overhead and comparing it to other proposed schemes. It is compared with the group-based handover control scheme for mobile internet using partially distributed mobility management (GP-DMM) [42] and a secure blockchain-based group mobility management scheme in the VANET (SEBGMM) [43]. The methodologies employed for the calculation of signaling overhead within the framework of GP-DMM are delineated in the subsequent equation [42].

(14) $$\begin{array}{cc} C_{DMM}= & K[a\left(L_{Rs}+L_{RA}\right)hop_{\left(v-RSU\right)}+2b\left(L_{PBA}+L_{PBU}\right)hop_{\left(CMD-RSU\right)} \end{array}$$

The signaling overhead associated with SEBGMM for K Vs’ handover is demonstrated as follows [43]:

(15) $$\begin{array}{cc} C_{SEGBMM}= & K[a\left(L_{Rs}+L_{RA}\right)hop_{\left(v-RSU\right)}] \end{array}$$

**Figure 10:**
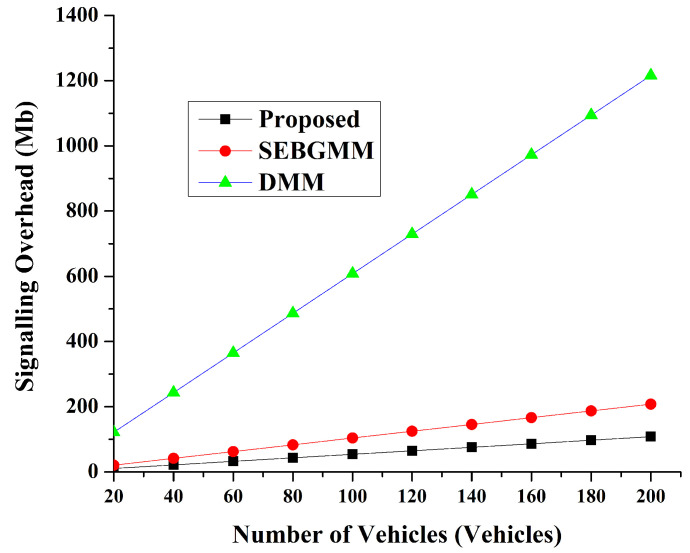
Signalling overhead.

The signaling overhead for this proposed system (KBC) is shown as follows:

(16) $$\begin{array}{cc} C_{KBC}= & hop_{RSU-RSU}\left[a\times Trans_{u}\times L_{msg}\right] \end{array}$$

where $hop_{RSU-RSU}$ represents the mean spatial separation between two Roadside Units (RSUs), *a* symbolizes the coefficient of weighting assigned to a particular link, $Trans_{u}$ signifies the unit of transmission, and $L_{msg}$ indicates the aggregate size of the message that is exchanged during the signaling procedure. The size of the selected messages in the signaling process in this study is delineated in Table 6, while the comparative outcomes with prior research are illustrated in Figure 10. All the schemes used blockchain-based security in VANETs. The initial methodology (GP-DMM) [42] exhibits the most substantial signaling overhead, attributable to its framework that inadequately addresses session key negotiation and the preliminary authentication phase of nodes. Our proposed framework and SEBGMM [43] exhibit parallels in differentiating between the initial authentication and handover phases. Nonetheless, the proposed framework demonstrates the minimal signaling overhead attributable to its more streamlined architecture, which does not employ a Control Mobility Database (CMD) and solely relies on the TAS and RSU.

### 4.3. Blockchain Performance Results

The four main functions of the smart contracts that make up the proposed system are as follows: Deployment, EntityRegistration(), AuthenticationMessageUploading(), and AuthenticationMessageAccessing(). The Ethereum blockchain platform compiles and deploys these contracts, determining how much gas is needed for each operation. Table 7 presents the basic gas consumption for each smart contract operation in our proposed system. To calculate the basic gas consumption, first, one user registered and then uploaded an authentication message to the block of the blockchain. After that, another user accessed this authentication message from the block. To do so, the gas values for every operation were calculated. The experimental results indicate that, out of these four basic operations, the maximum gas usage for the Deployment operation is GWEI 1,145,042, significantly lower than the block gas limit of GWEI 6,721,975, demonstrating the practicality of our system.

The transaction costs in Ethereum correlate with gas prices, with a baseline of GWEI 1 equating to $10^{-9}$ Ether. Throughout the analysis period, on 10 February 2024, the Ethereum to US dollar exchange rate stood at 1 Ether = USD 2487.23. Initially, the smart contract’s deployment cost is approximated. This preliminary cost is essential for system initialization. Following this, the costs of execution for various smart contract operations are computed. The corresponding transaction cost of basic gas consumption by the system is also listed in Table 7. This analysis demonstrates that the proposed strategy is practical for real-world implementation, considering the system’s transaction costs [44].

**Figure 11:**
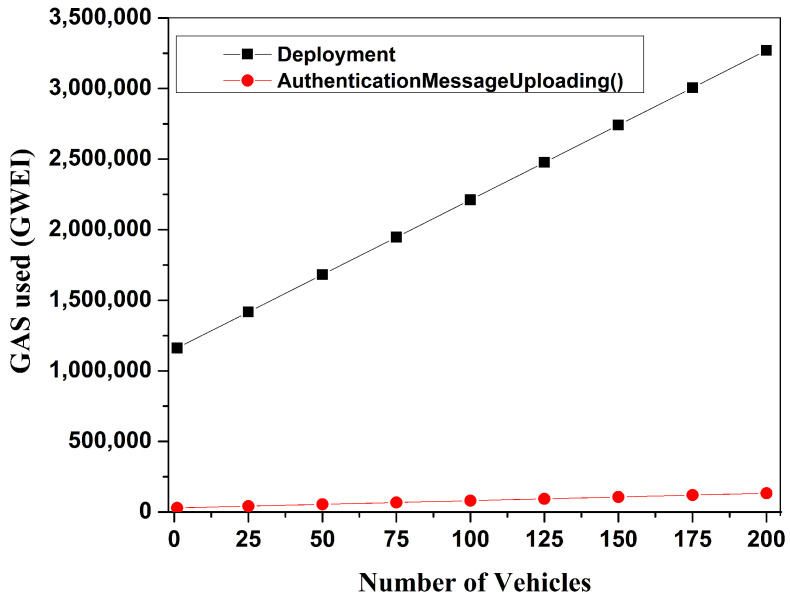
Number of vehicles vs. gas values.

We also analyzed the impact of the quantity of vehicles on the system’s gas values. In this study, we varied the number of vehicles from 1 to 200 and calculated the corresponding gas values for several operations. Increasing the number of vehicles means more authentication messages must be stored in the blockchain block, affecting the gas values for the Deployment and AuthenticationMessageUploading() operations, as these two operations depend on the number of authentication messages. Figure 11 depicts this variance by showing that when the number of vehicles grows from 1 to 200, the gas value increases linearly. The study also highlighted the system’s practicality, with the highest required gas value being GWEI 3,270,774 to execute the Deployment operation for 200 vehicles, which is well below the block’s maximum gas limit of GWEI 6,721,975.

**Figure 12:**
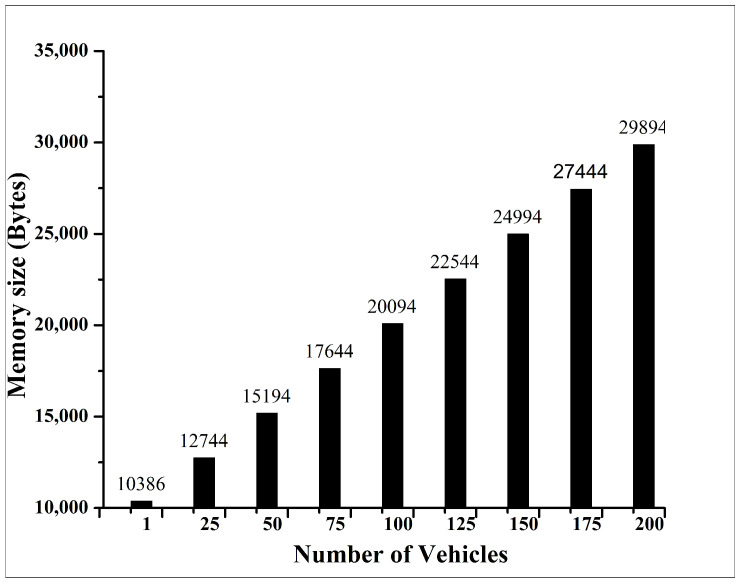
Memory size required for the block to store various authentication message.

The overall message size is crucial for effective message transfer communication in the VANET system. However, the size of an authentication message in a VANET system varies depending on the authentication protocol used. In general, the message size can range from hundreds of bytes to a few kilobytes, depending on the cryptographic algorithm, security features, and the specific VANET application. In [45], the authors used the message size ranges from 50 to 1200 bytes for secure messaging in the VANET system. In our study, we used the authentication message size of 32 bytes (without encryption), and we achieved an authentication message size of 48 bytes (after executing AES-128 encryption). The Ethereum blockchain regulates the size of blocks using the concept of gas, which calculates the amount of memory and processing time required for a transaction. The block gas limit sets a cap on the total gas within a block, which indirectly determines data storage capacity. Increasing the gas limit allows for more data to be included in a block [46]. Our investigation focuses on the storage of up to 200 vehicles, each identified by one authentication message. The memory needed to execute the transactions and to store in a block with the varying number of authentication messages is illustrated in Figure 12. This illustration demonstrates the relationship between authentication messages and memory storage in a block. Our experiment used the typical Ethereum block size of 1–2 MB [47]. When the transaction in blockchain is successfully completed, storing an authentication message in the blockchain network needed approximately 10 kilobytes of memory. The maximum memory required to store 200 authentication messages (for 200 vehicles) was found to be approximately 30 kilobytes, which is significantly lower than the maximum block size (1–2 MB) of the Ethereum network. This evaluation underscores the practicality of the system in terms of memory demands by the system.

### 4.4. The Security Analysis

The authentication messages are rendered immutable in the blockchain due to cryptographic hashing, whereby each block contains a unique hash derived from the previous block’s data, creating an interlinked chain. This proposed system used the Ethereum blockchain that utilizes SHA-3 for its hashing operations. The inherent properties of cryptographic hash functions, such as collision resistance and irreversibility, contribute to the prevention of authentication message tampering or deletion. Furthermore, the Proof-of-Work (PoW) agreements method ensures agreement among the RSUs and the TAS on the validity of transactions before adding the authentication message to the blockchain. This decentralized agreement mechanism fortifies resistance against malicious alterations by requiring a majority consensus, making the authentication system robust and resilient against unauthorized modifications, thus ensuring the integrity and security of authentication for our proposed system.

The integration of blockchain with Kerberos authentication in this proposed system introduces a noteworthy enhancement in privacy preservation by the implementation of smart contracts. It creates agreements or conditions between the RSUs and TASs. This condition must be fulfilled by the entities in order to perform the transaction. In our proposed system, the smart contract can automate the entities’ registration, uploading the authentication message and requesting it. With the smart contract, we set that only the TAS entity can make an AuthenticationMessageUploading() request. On the other hand, RSU entities are able to make AuthenticationMessageAccessing() requests. This selective access ensures that only authenticated and authorized entities can access specific information, mitigating the risk of data exposure to unauthorized parties. Consequently, the use of smart contracts reinforces the privacy preservation entity by restricting access to sensitive information to only those entities that meet the predefined criteria, enhancing the security and reliability of the authentication framework within the context of vehicular communication.

The verification process to add a new block involves all of the network users, including RSUs and TASs, to ensure a collective responsibility for transaction execution, engendering a heightened level of trust among participants. The decentralized nature of blockchain mandates a PoW consensus mechanism, requiring agreement from the majority before transactions are added to the distributed ledger. This distributed validation mechanism contributes to the transparency of the network, as all transaction records are permanently inscribed in the blockchain. This transparency fosters accountability and fortifies the integrity of the authentication system by enabling all users to verify transaction history. Consequently, the usage of blockchain in this proposed system augments trust through shared responsibility and fosters transparency by recording transactions on an immutable and accessible ledger, thereby bolstering the reliability of the authentication processes within vehicular communication.

In the VANET system, malicious vehicles enter themselves into communication between two vehicles, impersonating them to gain access to information and inject false data. This type of attack is known as a man-in-the-middle attack, where the attacker eavesdrops on communication, alters messages, and breaches data integrity and privacy goals. The attacker may successfully pass through user authentication but will be blocked at the possession approval step. By inserting false information between genuine nodes or vehicles, the attacker undermines the trustworthiness of the data being exchanged, compromising network security. This attack poses a significant threat to the security standards of vehicle communication systems [48]. Due to the integration of blockchain with VANETs within the suggested framework, both the sender and the recipient must follow several procedures to complete the transaction successful. It does not need the assistance of man-in-the-middle to carry out the steps, even though passing them is necessary. A smart contract contains the predetermined terms of the transaction. These terms are automatically evaluated and validated throughout the transaction. This mitigates the man-in-the-middle attack. The integration of Kerberos authentication in VANETs enables mutual authentication between vehicles and RSUs, meaning both parties authenticate each other through the Key Distribution Center (KDC). By ensuring that both sender and receiver are legitimate, Kerberos makes it more challenging for an attacker to impersonate either party. Moreover, we use AES-128 symmetric key encryption to generate a session key for each communication session. This session key is known only to the authenticated parties, reducing the risk of an attacker intercepting and decrypting messages.

A consensus mechanism in a blockchain technology is a fault-tolerant mechanism that is advantageous to a single state of the network among the distributed multi-node systems in achieving the required agreement. The agreement is a list of rules and regulations for all the different participating nodes, which will eventually be helpful in deciding their contributions. Moreover, any transactions or events that take place in the system will be updated from time to time in the blockchain, and all the nodes will notify it. So, it is hardly possible to doubt the transparency of the transactions in a network that ultimately creates the trust among the nodes [39]. The Denial of Service (DoS) attack is an attack on the availability of the network. The main purpose of this attack is making the network unavailable to legitimate nodes. The attacker node generates a high volume of network traffic and consumes all the bandwidth of the network and makes it impossible for the RSU to manage such high-volume traffic, due to which the network become unavailable to the nodes [49]. Due to the characteristics of blockchain, Denial of Service (DoS) attacks do not seem to affect the system, as the consensus mechanism only allows legitimate nodes to participate in the network [39]. Kerberos relies on a Ticket Granting Ticket (TGT) to streamline session authentication. Once the initial authentication is complete, the TGT allows vehicles to access network services without contacting the AS each time. This minimizes bandwidth usage and decreases the number of authentication requests, therefore reducing the load on the network and limiting opportunities for DoS attacks.

### 4.5. The Scalability Challenges

In the design and implementation of a Kerberos-blockchain VANET authentication system, scalability is a critical consideration, especially when applied across diverse network scenarios such as suburban and urban environments. As the number of vehicles and number of TASs increases, the system must efficiently manage the load distribution of the TASs. Furthermore, while the blockchain component of the system is designed to store authentication messages, the block size limitation becomes a significant factor.

As the number of vehicles and TAS increases within the network, ensuring an even and efficient load distribution among TASs becomes a crucial challenge. In the suburban scenario, the number of vehicles is set to 100, whereas it increases to 200 in the urban environment for scenario 2, with the same number of TASs. Since scenario 2 did not meet the VANET requirements concerning the maximum authentication delay, we increased the number of TASs to two in scenario 3. This increase resulted in a reduction in the authentication delay, bringing the delay below the 100 ms threshold, thereby fulfilling the VANET criteria. However, the reduction in delay from scenario 3 is not exactly 50%, despite the addition of a second TAS, but is instead 40.3%. This discrepancy is influenced by the Ad-Hoc On-Demand Distance Vector (AODV) routing protocol, which selects the shortest path based on the node count between the source and the destination. When the network includes two TAS units, the load may become imbalanced, as each TAS may have different node counts between itself and the vehicles. This imbalance can lead to one TAS becoming more heavily loaded than the other, resulting in higher authentication delays for the vehicles managed by that TAS. As the vehicle density increases and one TAS handles more load than the other, there is a potential risk of bottlenecks and performance degradation if the load is not managed properly. To address the load imbalance issue, several protocols could be considered for future work, such as the Dynamic Load Balancing Protocol (DLBP), Least Loaded Server Protocol, Round-Robin Load Balancing, or other load balancing methods. However, the results of this study indicate that the proposed method did not cause any bottlenecks, as the performance of scenario 3 satisfied the VANET delay requirements.

Another scalability challenge in the proposed method is the block size limitation for storing authentication messages. The maximum block size in Ethereum ranges from 1 to 2 MB. It is critical that the messages stored within each block do not exceed this limit, as doing so may increase the risk of blockchain forking and result in inconsistencies across the network. To assess the impact of vehicle density, we evaluated the effects of increasing the number of vehicles from 100 to 200 in our experiments. The maximum storage required for 200 authentication messages was calculated to be only 29.894 KB, which is significantly below the maximum block size threshold. Therefore, even as scalability expands, the three scenarios presented in this study remain well within the acceptable block size limits.

### 4.6. Comparative Analysis

The proposed system is also compared with some of the existing literature. Table 8 summarizes such comparison in terms of vehicle capacity, encryption techniques, Kerberos integration, gas, cost, delay parameters, and security attacks analysis.

The variation of the number of vehicles indicates the scalability of the system, which can influence the network topology and the effects of network performances in denser topology. In the literature, refs. [12,14,15,16,50,51] evaluated the parameters of the system considering the maximum number of vehicles to be 200 for the city area. Based on this, to evaluate the performance of our system we considered the number of vehicles from 1 to 200.

The exact number of vehicles in the VANET system can vary based on factors such as the size of the area, specific use case (e.g., safety, traffic management), and the simulation’s goals. Denser urban areas often have higher vehicle counts, while suburban or less populated areas use lower values. In practice, the number of vehicles is chosen based on a balance between real-world traffic patterns and computational resources for the simulation.

By incorporating Kerberos, VANET systems benefit from improved security through efficient, scalable, and tamper-resistant authentication protocols. Kerberos is a secure protocol that enables mutual authentication between entities, reducing the risk of impersonation attacks. It uses a ticket-granting mechanism, requiring only one communication with the Key Distribution Center (KDC) for a ticket. Kerberos timestamps each ticket, preventing reuse by malicious actors. It supports large networks with multiple entities, making it ideal for VANET environments with numerous vehicles and infrastructure nodes.

In our proposed system, we used the AES-128 encryption technique to encrypt the authentication message. This brings several distinct benefits instead of using ECC encryption, which is used by most of the literature [14,15,16,50,51], except for [13], which also considers AES encryption. AES is a faster, simpler, and resource-efficient symmetric encryption method compared to ECC. It uses the same key for both encryption and decryption, making it ideal for real-time applications in VANETs. Additionally, AES requires less computational power, making it suitable for low-power devices and real-time traffic updates.

The integration of blockchain with VANET requires careful analysis of gas and cost implications. The gas values also indicate the real cost that will be spent to execute the system. Gas value analysis can be beneficial for companies considering using a blockchain-based system. Optimizing smart contract code is crucial for real-world applications. A lightweight design reduces computational burden, making it viable for real-time operations in VANETs. Unlike most of the literature [12,14,15,50,51], our system analysis of gas and cost implications demonstrates its practicality.

Fast authentication is crucial in VANETs due to the high mobility of vehicles. Minimizing authentication delay ensures secure access and rapid information exchange. High handover delay can cause communication interruptions or network disconnections. Reducing end-to-end delay is essential for real-time communication. Our literature has analyzed all three possible delays (authentication delay, handover delay, and end-to-end delay) to ensure practicality, while some studies [12,15,50] focus only on end-to-end delay, and some others [13,16] investigate only authentication delay.

## 5. Conclusions

This study introduced a blockchain and Kerberos-based authentication framework for VANETs, which encapsulated authentication messages within blockchain blocks. The practicability of implementing blockchain technology and the network’s performance were evaluated. We executed three experiments to test the applicability of the system in diverse network scenarios to fulfill network and blockchain requirements. In the first scenario, we tested the proposed system in a suburban area with 100 vehicles and 1 TAS. In the second scenario, we changed the environment to an urban area that had an increased number of vehicles of 200, without any TAS addition. In the third scenario, we used the second scenario environment with one TAS addition. The performance of the system was assessed through network performance and blockchain performances impacted by TAS and the number of vehicles. Network performance included authentication, handover, and end-to-end delays. The blockchain performances included the gas value and the block size. The study revealed that, in the first scenario, the system fulfilled all of the network requirements. However, in the second scenario it did not fulfill the authentication delay requirement of VANETs because it exceeded the maximum limit of 100 ms. The third scenario overcame that authentication delay problem by increasing the number of TASs to two. Concerning blockchain practicability, although the gas value and memory size of the system rose linearly with vehicle quantity, the system remained practicable as it did not exceed the maximum allowable gas value and memory size of the block. All of the findings showed that our proposal is applicable to be implemented in diverse network scenarios with the addition of a TAS. In future work, we would like to vary the vehicle’s communication range in the system, which can reduce broadcast-storm and frequent disconnections when the vehicular density is high.

## Figures and Tables

**Table 1:** Comparative study of proposed literature with some existing authentication for VANET literatures.

Reference/Year	Security Methods	Blockchain Utilization	Simulation Environment	Evaluation Area	Evaluated Parameters
Wang et al. [10]/2020	CA decentralization and biological-password-based	No	One (Opportunistic Networking Environment) and ProVerif	Urban area (ring road of Beijing)	Performance evaluation including authentication overhead and certificate updating overhead
Wang et al. [11]/2021	Pseudonym-based and group-based authentication algorithm	No	ns3, Sumo, and MIRACL	Urban area (Guilin city)	The study analyzes RSU computation and communication performance, service capabilities, and v2v authentication
Lee et al. [12]/2021	Honeylist technique, SHA-256, and XOR operation	No	NS3	not indicated	It considers factors such as end-to-end delay, throughput, computation cost, communication cost, and energy consumption cost
Gürfidan et al. [13]/ 2023	AES, TKIP, and CCMP encryption and blockchain	Yes	Hyperledger fabric and apache jMeter	Not indicated	Evaluated performance based on authentication time
Li et al. [14]/2023	ECC and blockchain	Yes	Hyperledger Fabric	Not indicated	It evaluates the performance of BDRA, including simulation parameter setting, theoretical computation time cost, and stability validation
Lee et al. [15]/2022	Blockchain and ECC	Yes	NS3 and BAN logic	not indicated	The performance analysis considers computational cost, end-to-end delay, and throughput
Sang et al. [16]/2023	Blockchain and ECC	Yes	NS2	not indicated	It considers computational overhead, authentication delay, transaction latency, and gas consumption
Rahayu et al. [Proposed]/2024	Kerberos authentication, AES encryption, and blockchain	Yes	Omnet++, Sumo, Truffle, and Ganache	Suburban and urban area	It considers authentication delay, handover delay, end-to-end delay, gas consumption, and memory size of the blockchain

**Table 2:** Annotations and abbreviations.

Entities		
Vehicular Ad-Hoc Network		VANET
On-Board Unit		OBU
Vehicle		V
Trusted Authority Server		TAS
Authentication Server		AS
Ticket Granting Server		TGS
Roadside Unit		RSU
Keys		
Vehicle’s secret key		$K_{Vs}$
TGS secret key		$K_{TGSs}$
TGS session key		$K_{TGSse}$
Service session key		$K_{Sse}$
Service secret key		$K_{Ss}$
Signature of the Client		$P_{v}$
Signature KDC		$P_{kdc}$
Certificate KDC		$C_{kdc}$
Name of Message		
General	Request authentication	RA
	Reply	RP
	Ticket Granting Ticket	TGT
	Service Ticket	ST
Authentication msg	Vehicle auth V to TGS	$AU_{VtTGS}$
	Vehicle auth V to Service	$AU_{VtS}$
	Service auth message	$AU_{Sm}$
	Service authentication Message	FA
Attributes	Attributes AS to V	$AT_{AStV}$
	Attributes V to TGS	$AT_{VtTGS}$
	Attributes TGS to V	$AT_{TGStV}$
Message Contents		
ID	Vehicle’s ID	$ID_{v}$
	Service name/ID	$ID_{S}$
	TGS name/ID	$ID_{TGS}$
IP	User IP Address	$IP_{v}$
Req	Request lifetime for TGT	$Req_{TGT}$
	Request lifetime for ticket	$Req_{LT}$
Timestamp	Timestamp attributes AS to V	$TS_{AStV}$
	Timestamp TGT	$TS_{TGT}$
	Timestamp Vehicle authentication	$TS_{VA}$
	Timestamp attribute TGS to V	$TS_{TGStV}$
	Timestamp ST	$TS_{ST}$
	Timestamp vehicle to service	$TS_{VtS}$
	Timestamp service auth message	$TS_{StV}$
Parameter	DH parameter	$DH_{p}$
Lifetime	Lifetime for TGT	$LT_{TGT}$
	Lifetime for Service Ticket	$LT_{ST}$

**Table 3:** Implementation environment.

Software	Configuration/Version
OS	Windows 11 22H2 64-bit
CPU	AMD Ryzen 7 5800U CPU @ 1.90 GHz
RAM	16 GB
Truffle	5.11.0
Ganache	2.7.1
Omnet++	6.0.2
SUMO	1.4.0

**Table 4:** Simulation properties.

Parameters	Units of Type
Communication model	IEEE 802.11p
Simulation area	5000 m × 2500 m
Communication range of RSU	1000 m
Communication range of vehicle	100 m
Data rate	27 Mbps
Safety messages	Every 30 s
Mobility	Duaroute Mobility

**Table 5:** Network requirements fulfillment resume.

Scenario	Authentication Delay	Handover Delay	End-to-End Delay
1	✔	✔	✔
2	×	✔	×
3	✔	✔	✔

**Table 6:** The size of messages in the signalling process.

Parameters	Value
Session Key	16 byte
Vehicle ID	8 byte
TS	4 byte
Ticket for initializing authentication	8 byte
HMAC	8 byte
Input bit length AES	16 byte
Lifetime	3 byte
IP Address	16 byte
Service Name	3 byte

**Table 7:** The gas used for several operations of smart contracts.

Operation	Gas Consumption (GWEI)	Tx Cost (Ether)	Tx Cost (in USD)
Deployment	1,145,042	0.001145	2.8478
EntityRegistration()	112,223	0.000112	0.2785
AuthenticationMessageUploading()	29,733	0.000029	0.0721
AuthenticationMessageAccessing()	27,208	0.000027	0.0671

**Table 8:** Comparison of proposed method with existing literature.

Reference/Year	Vehicles for Simulation	Kerberos Integration	Encryption Technique	Gas and Cost Calculation	Delay Evaluation	Security Attack Analysis
[12]/2020	25–50	×	not defined	×	Yes, end-to-end delay	×
[13]/2020	not defined	×	AES and CCMP	✔	Yes, authentication delay	✔
[14]/2020	1–50	×	ECC	×	Not Evaluated	✔
[15]/2022	10–50	×	ECC	×	Yes, end-to-end delay	✔
[16]/2023	25–200	×	ECC	✔	Yes, authentication delay	✔
[50]/ 2018	1–50	×	ECC and IBE	×	Yes, end-to-end delay	✔
[51]/2024	20–100	×	ECC	×	Not evaluated	✔
Proposed/2024	1–200	✔	AES-128	✔	Yes, authentication delay, handover delay, and end-to-end delay	✔

## Data Availability

Data are contained within the article.
